# Inhibition of PirB Activity by TAT-PEP Improves Mouse Motor Ability and Cognitive Behavior

**DOI:** 10.3389/fnagi.2017.00199

**Published:** 2017-06-20

**Authors:** Ya-Jing Mi, Hai Chen, Na Guo, Meng-Yi Sun, Zhao-Hua Zhao, Xing-Chun Gao, Xiao-Long Wang, Rui-San Zhang, Jiang-Bing Zhou, Xing-Chun Gou

**Affiliations:** ^1^Institute of Basic and Translational Medicine, and School of Basic Medical Sciences, and Shaanxi Key Laboratory of Brain Disorders, Xi’an Medical UniversityXi’an, China; ^2^Department of Anesthesiology, Tangdu Hospital, Fourth Military Medical UniversityXi’an, China; ^3^Department of Neurosurgery, School of Medicine, Yale UniversityNew Haven, CT, United States

**Keywords:** PirB, motor capacity, cognitive behavior, TAT-PEP, BDNF

## Abstract

Paired immunoglobulin-like receptor B (PirB), a functional receptor for myelin-associated inhibitory proteins, plays an important role in axon regeneration in injured brains. However, its role in normal brain function with age has not been previously investigated. Therefore in this study, we examined the expression level of PirB in the cerebral cortex, hippocampus and cerebellum of mice at 1 month, 3 months and 18 months of age. The results showed that the expression of PirB increased with age. We further demonstrated that overexpression of PirB inhibited neurite outgrowth in PC12 cells, and this inhibitory activity of PirB could be reversed by TAT-PEP, which is a recombinant soluble PirB ectodomain fused with TAT domain for blood-brain barrier penetration. *In vivo* study, intraperitoneal administration of TAT-PEP was capable of enhancing motor capacity and spatial learning and memory in mice, which appeared to be mediated through regulation of brain-derived neurotrophic factor (BDNF) secretion. Our study suggests that PirB is associated with aging and TAT-PEP may be a promising therapeutic agent for modulation of age-related motor and cognitive dysfunctions.

## Introduction

Paired immunoglobulin-like receptor B (PirB) is a functional receptor for myelin-associated inhibitory proteins, including Nogo, myelin-associated glycoprotein (MAG) and oligodendrocyte-myelin glycoprotein (OMgp), which inhibit axonal regeneration and functional recovery after brain injury (Gou et al., 2014). The expression level of PirB in neurons has been shown to increase after neurological injuries, including spinal cord injuries (Zhou et al., 2010), hypoxic-ischemic brain damage (Adelson et al., 2012; Wang et al., 2012; Guo et al., 2013), encephalitis (Deng et al., 2012), hippocampal aging (VanGuilder Starkey et al., 2012) and retinopathy (Cai et al., 2012). Blocking PirB activity through antibody antagonism or genetic approaches allowed the promotion of axon regeneration and synapse plasticity (Adelson et al., 2012; Wang et al., 2012; Kim et al., 2013; Bochner et al., 2014).

Recent emerging evidence further suggests that PirB may regulate cognitive functions in addition to neurite regeneration. It was found that the expression of PirB in the cortex and hippocampus was significantly upregulated in rats with memory deficits induced by lipopolysaccharide (Deng et al., 2012). The expression of PirB was also found to gradually increase in the CA1 and DG subregions of the hippocampus in aging mice with cognitive behavior deficits (VanGuilder Starkey et al., 2012). Blocking PirB receptor could enhance both the short-term and long-term cognitive functions after bilateral common carotid artery occlusion in mice (Deng et al., 2016; Li et al., 2017). However, neither the association between PirB and cognition in normal brain development nor whether PirB can be used as a pharmacological target for modulating cognitive functions has been previously studied.

In this study, we examined the pattern of PirB expression in the brain of mice at different developmental stages. We found that the expression of PirB in the cerebral cortex, cerebellum and hippocampus increased with age. Then, we explored whether PirB could be targeted to improve age-related cognitive dysfunctions by using TAT-PEP, a recombinant protein consisting of soluble PirB ectodomain fused with TAT. TAT is a cell penetration peptide derived from the TAT-protein in the human immunodeficiency virus and was previously successfully used for facilitating drug delivery to the brain by us and others (Aarts et al., 2002; Wang et al., 2008). We demonstrated that treatment with TAT-PEP significantly increased the length of axons in neurons *in vitro* and improved exhaustive swimming capacity, spatial learning and memory in mice. Our study suggests that PirB plays an important role in aging and is a promising target for pharmacological modulation of cognitive function.

## Materials and Methods

### Animals

All animal procedures were approved by the Animal Care and Ethical Committee at Xi’an Medical University (Permit Number: 2012-8, 7 March 2012). Male C57BL/6 mice of 1 month, 3 months and 18 months were supplied by the Experimental Animal Center of Xi’an Jiao Tong University. Efforts were made to reduce the number of animals used in the study by following the 3Rs (reduction, refinement and replacement). The total number of mice used in our experiments was 180.

### Construction, Expression and Purification of TAT-PEP

TAT-PEP (PirB extracellular peptide) construction, expression and purification were carried out as previously reported (Deng et al., 2016). Briefly, cDNA of extracellular domain of PirB was synthesized and cloned into expression vector pTAT-HA-6xHis. For protein expression, pTAT-PEP was transformed into BL21 (DE3). Protein production was induced with 100 mM isopropyl β-D-1-thiogalactopyranoside (TaKaRa, Tokyo, Japan), and purified by Ni-NTA-agarose chromatography (Merck, Darmstadt, Germany). The size and purity were confirmed by sodium dodecyl sulfate-polyacrylamide gel electrophoresis (SDS-PAGE). As a control peptide, the scrambled PEP fusion protein named as TAT-mPEP was expressed and purified according to the same procedure used for TAT-PEP (Deng et al., 2016).

### Overexpression PirB in PC12 Cells

PC12 cell lines derived from rat adrenal gland pheochromocytoma were cultured in Dulbecco’s Modified Eagle’s Medium (DMEM) with 10% horse serum, 5% FBS and 1% penicyline/streptomycine at 37°C and 5% CO_2_. Cells with overexpressed PirB were obtained through lentiviral transduction of mouse PirB, which was constructed by HANBIO company (Shanghai, China), and then selected with puromycin (8 μg/ml) according to our recently published procedures (Chen et al., 2015). These cells were named PC12^PirB^ cells.

### Determination of the Length of Axons

PC12 cells and PC12^PirB^ cells were plated on glass coverslips, and one half of PC12^PirB^ cells were treated with 150 μg/L TAT-PEP. After 24 h, cells were stained with the anti-β-tubulin antibody (Catalog#: MA5-16308, 1:200, Thermo Scientific) and then the Alexa-594-labeled donkey anti-mouse IgG secondary antibody (1:800, Thermo Scientific). Cells were imaged using a fluorescence microscope (OLYMPUS IX73). The length of an axon was determined as the linear distance from the point of exit to the end of the longest branch of the neurite according to a previous report (Richardson et al., 2007). In every experimental group, 150–200 cells were analyzed.

### Design of Animal Studies

A scheme for animal studies was shown in Figure 4A. Mice at the selected ages were randomly divided into two groups, with 12 mice in each group. One group received treatment of TAT-PEP in saline, another group received TAT-mPEP in saline as control. Treatments were carried out through intraperitoneal administration of TAT-PEP or TAT-mPEP at 8 mg/kg/injection, twice a day, for 30 or 60 days, as indicated in Figure 4A. Behavior training and evaluation were performed according to the time points indicated in Figure 4A.

**Figure 1:**
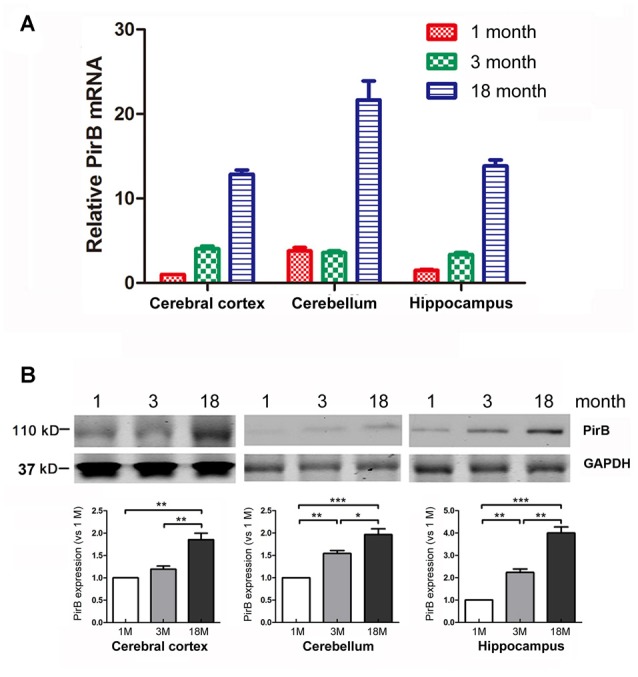
Quantification of the expression of paired immunoglobulin-like receptor B (PirB) in mouse brains (*n* = 3). Expression of PirB at the mRNA level **(A)** and protein level **(B)** in the cerebral cortex, cerebellum and hippocampus of 1 month, 3 months and 18 months mice were quantified using Quantitative Real-time PCR (qPCR) and western blot respectively. *p < 0.05, **p < 0.01, ***p < 0.001.

**Figure 2:**
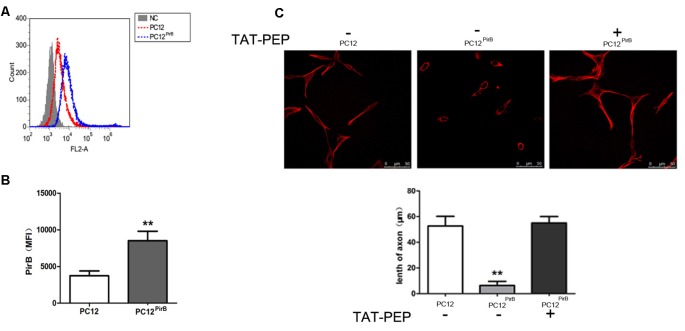
Overexpression of PirB inhibited neurites outgrowth in PC12 cells, which was reversed by TAT-PEP treatment (*n* = 4). Flow cytometry diagram **(A)** and quantification **(B)** of PirB expression in PC12^PirB^ cells (NC means normal PC12 cells without any treatment). **(C)** Representative images of PC12 cells, PC12^PirB^ cells with and without treatment of TAT-PEP. Cells were stained with anti-β-tubulin antibody. The length of axons was measured using fluorescence microscope. **p < 0.01.

**Figure 3:**
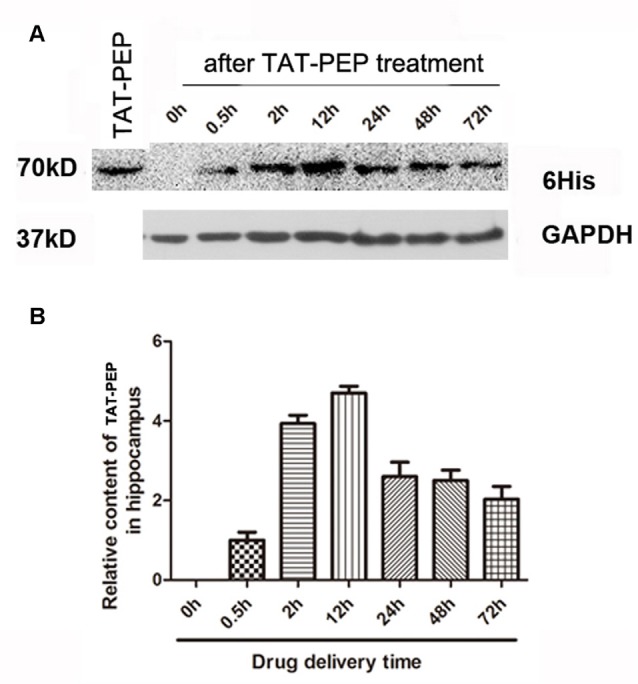
TAT-PEP penetrated the brain after intraperitoneal injection (*n* = 4). Blot **(A)** and quantification **(B)** of TAT-PEP in the hippocampus at the indicated time points after intraperitoneal injection. The first lane was the sample of TAT-PEP protein provided as the positive control.

**Figure 4:**
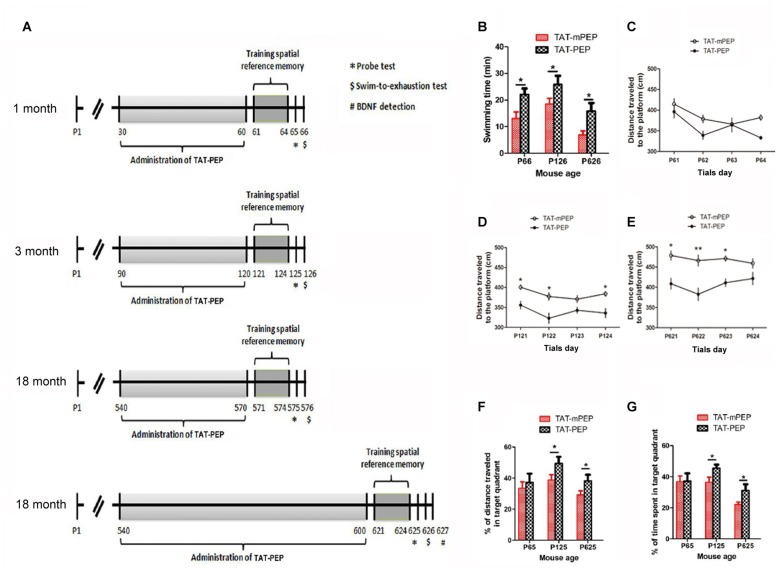
Intraperitoneal administration of TAT-PEP enhanced mouse motor capacity, spatial learning and memory (*n* = 12). **(A)** Schemes of experimental design for mice of different ages. Mice in 1 month, 3 months and the first 18 months groups received injection of TAT-PEP or TAT-mPEP for 30 days. In the second 18 months group, TAT-PEP or TAT-mPEP was given for 60 days. Spatial reference memory training in Morris water maze was performed at P61-P64 (1 month), P121-P124 (3 months), P571-P574 (the first 18 months group) and P621-P624 (the second 18 months group). Probe tests (*) were performed on the next day after the training. Swiming-to-exhaustion test ($) was performed at P66 for 1 month mice, P126 for 3 months mice, P576 for the first 18 months group mice and P626 for the second 18 months group mice. Mice in the second 18 months group were sacrificed at P627 for ELISA analysis of brain-derived neurotrophic factor (BDNF). **(B)** Evaluation of exhaustive swimming capacity was performed in the next day of Probe test. Mice were forced to swim until they were exhausted. The length of swimming time was recorded. **(C–E)** The distance traveled to the platform of different age mouse was recorded in the training of the spatial reference memory task in the Morris water maze. **(F,G)** The spatial learning and memory were measured by Probe test using water maze. Percentage of distance in the target quadrant is relative to the total distance in the water maze **(F)** in indicated groups, and percentage of time in the target quadrant is relative to the total time in the water maze **(G)**. During the above experiments **(B–G)**, there was no significant difference between the 18 months group received TAT-PEP and TAT-mPEP for 30 days. Data was not shown. *p < 0.05, **p < 0.01 for mice with TAT-PEP treatment compared with TAT-mPEP treatment.

### Morris Water Maze

Water maze tests were performed as previously described (Torres et al., 2015). The diameter and the depth of the circular pool were 90 cm and 50 cm, respectively. The inner wall was carefully cleaned to eliminate any local cues. The temperature of the room and water was kept at 23 ± 1°C, and the pool was filled with water to a depth of 40 cm and rendered opaque by the addition of milk to hide the escape platform. The plexiglas platform of 9 cm in diameter was placed in the pool 1 cm below the water surface. The recording camera was connected to a digital tracking device, and the water-maze software was used to process the tracking information. The platform position remained constant throughout the training period.

In the training period, each animal performed four trials per session, one session per day, for 4 days. The intertrial interval (ITI) was 1 min. During each session, the mouse started from a random position in the pool, and the maximum time allowed for swimming and searching was 60 s. Mice were allowed to remain on the platform for 10 s after they had found it. The subjects that failed to find the platform were gently guided to it and placed on the platform for 10 s. The average distance traveled to the platform per day was recorded. On the fifth day, probe test was performed with the platform removed from the pool. Mice were allowed to swim for 60 s. The distance traveled and the time spent in target quadrant were recorded, and the percentages were calculated from the recorded data.

### Swimming-to-Exhaustion Test

On the next day after the probe test, a weight (5% body weight) was attached to the tail of each mouse for the swim-to-exhaustion test (You et al., 2015). The swimming exercise was carried out in a tank filled with water in 38 cm depth and at 34 ± 1°C. Mice were defined to be exhausted when they failed to rise to the surface of the water to breathe within 5–7 s period. Swimming time was recorded for each mouse.

### Quantitative Real-Time PCR (qPCR)

Total RNA was isolated using the Trizol reagent (Invitrogen), and treated with RNase-free DNase I (Roche) to remove residual DNA. cDNA was obtained using ReverTra Ace quantitative real-time PCR (qPCR) RT Kit (TOYOBO). qPCR was carried out using ABI Stepone plus and Realtime PCR Master Mix (SYBR Green; TOYOBO). Primers for mouse PirB were: 5′-TACAAGGAAGTACCACGCCC-3′ (forward) and 5′-GGTTCAGCCTTGATGGTTGG-3′ (reverse). GAPDH was used as the endogenous control.

### Western Blot

Proteins were prepared using RIPA lysis buffer containing protease and phosphatase inhibitors (Beyotime Biotechnology). Protein concentrations were determined using BCA protein assay (Thermo Scientific). Proteins were separated using 8% SDS-PAGE and transferred to the polyvinylidene fluoride membranes (Millipore). The membranes were blocked for 1 h, followed by incubation with the primary antibodies overnight, and then incubated with the HRP-conjugated anti-mouse antibody afterwards (1:10,000, Roche). The primary antibodies were anti-6xHis antibody (1:1000, Abcam) and anti-PirB (1:500, Thermo Scientific). GAPDH was used as loading control and determined using the anti-GAPDH antibody (1:2000, Santa Cruz). Blots were detected using BM Chemiluminescence Western Blotting kit (Roche). Densitometry quantification was analyzed using IPP6.0 software.

### Flow Cytometry

The cell surface expression level of PirB in transduced cells was determined using flow cytometry. Briefly, the cells were centrifuged and washed with phosphate buffered saline (PBS), followed by incubation with the primary antibody against PirB (1:200, Thermo Scientific) for 1 h on ice. After washing 3 times with PBS, the cells were treated with a 1:50 dilution of FITC-conjugated secondary antibody (1:800, Thermo Scientific). The cells were then washed 3 times with PBS and analyzed using a Accuri C6 flow cytometer (BD Biosciences), and data analysis was performed with Flowjo software.

### ELISA Assay

BDNF concentration was determined using the BDNF Emax ImmunoAssay (Promega, Madison, WI, USA) according to the manufacturer’s instructions. First, a 96-well microplate was sealed, incubated with anti-BDNF antibody (1:1000) overnight at 4°C, and washed with Tris-buffered saline (TBS). On the next day, the plate was blocked at RT for 1 h and washed. Samples and BDNF standards were added into the plate and incubated at RT for 2 h. After an extensive wash, the anti-BDNF antibody (1:500) was added to each well and the plate was incubated at RT for 2 h. After an additional wash, the HRP-conjugated anti-IgY (1:200) was added. One hour later, the plate was washed and incubated with TMB One solution for 10 min at RT. The reaction was ended by adding 1 M HCl. The absorbance was measured at 450 nm. The same procedures were used to determine the level of nerve growth factor (NGF).

### Data Analysis

**Figure 5:**
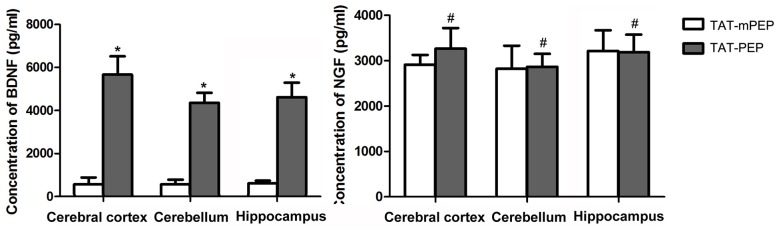
Quantification of BDNF and nerve growth factor (NGF) in 18 months mice with TAT-PEP treatment (*n* = 4). The level of BDNF and the level of NGF in the cerebral cortex, cerebellum and hippocampus of 18 months mice with 60-day TAT-PEP or TAT-mPEP treatment were examined using Emax ImmunoAssay System kits. Data were expressed in pg*ml-1. *p < 0.05, ^#^*p* > 0.05.

The data is expressed as the mean ± SD and analyzed with SPSS 13.0 statistical software. The data in Figures 4C–E was analyzed using two-way ANOVA with Bonferroni post-test analysis, with the treatments as the between-subject factor and the trial days as the within-subject factors. The data in Figures 1B, 2C were analyzed using One-way ANOVA with Dunnett’s test. The data in Figures 2B, 4B,F,G, 5 were analyzed using Student’s *t*-test.

## Results

### Expression of Endogenous PirB

To determine the temporal and spatial expression pattern of endogenous PirB in the brain, we harvested the cerebral cortex, cerebellum and hippocampus from mice at the age of 1 month, 3 months and 18 months. The expression of PirB in different regions was determined by qPCR and western blot. Results in Figure 1 showed that the expression of PirB increased gradually with age in all compartments at both the mRNA and protein levels, suggesting that PirB is associated with aging.

### Over-Expression of PirB Inhibits Axon Outgrowth, Which Can be Reversed by TAT-PEP Treatment

We studied the impact of PirB on axon outgrowth using PirB-overexpressed PC12 cells, PC12^*PirB*^, which were generated through lentiviral transduction. The results from the flow cytometry showed that the expression level of PirB in PC12^*PirB*^ cells was 2.4-fold of that in PC12 cells (Figures 2A,B). As shown in Figure 2C, overexpression of PirB significantly inhibits axon outgrowth. The average length of an axon in PC12^*PirB*^ cells is 7 μm, compared to 53 μm in control PC12 cells. This result is consistent with previous reports that PirB inhibits axon outgrowth (Gou et al., 2014; Liu et al., 2015).

Next, we investigated whether blocking PirB activity could reverse the axon outgrowth inhibition caused by PirB overexpression. TAT-PEP, a recombinant protein consisting of PirB extracellular motif fused with TAT, which demonstrated the ability to rescue neurite outgrowth inhibition induced by Nogo, MAG and OMgp in stroke (Deng et al., 2016), was used to treat PC12^PirB^. Results in Figure 2C showed that treatment with TAT-PEP effectively prolonged the growth of axon.

### TAT-PEP Penetrates the Brain

In our previous work, we demonstrated that the TAT peptide mediated efficient delivery of a 40 aa peptide, NEP1-40, to the brain (Han et al., 2016). To evaluate whether the conjugation of TAT enhances brain penetration of PEP, we injected TAT-PEP intraperitoneally into mice. At 0.5 h, 2 h, 12 h, 24 h, 48 h and 72 h after injection, the hippocampus was collected and processed. The presence of TAT-PEP was detected by western blot. Results in Figure 3 showed that TAT-PEP was detectable at 0.5 h after administration. The concentration of TAT-PEP in the hippocampus peaked at 12 h. The protein sample of the first lane was TAT-PEP synthesized *in vitro*, and here it was used as a positive control.

### Intraperitoneal Administration of TAT-PEP Enhances Mouse Motor Capacity, Spatial Learning and Memory

Motor deficit is one of the major results of aging. As the PirB expression is associated with aging, we set to study whether suppression of PirB activity by TAT-PEP could enhance mouse motor capacity. Based on the pharmacokinetic result described in Figure 3, TAT-PEP or TAT-mPEP was administered in mice every 12 h. Treatment lasted for 30 days for 1 month, 3 months and 18 months groups. Exhaustive swimming capacities in the three groups of mice were examined (Figure 4A). Compared to TAT-mPEP treatment, the time of swimming significantly increased in both 1 month and 3 months groups mice that received the 30-day TAT-PEP treatment (Figure 4B). With the same treatment regimen, the difference in the 18 months group between mice with TAT-PEP and TAT-mPEP treatments was insignificant (data not shown). However, the difference reached significance when the 60-day treatment was carried out (Figure 4B).

The spatial learning ability and memory in mice was examined using the Morris water maze evaluation method. Figures 4C–E showed the performance of both TAT-PEP and TAT-mPEP treated animals on the spatial reference memory tests. The group of 1 month mice injected with TAT-PEP exhibited no significant difference in the acquisition of the spatial reference memory task, as revealed by distance traveled to the platform in Figure 4C. For 3 months and 18 months groups, the TAT-PEP treatment notably increased the acquisition of the spatial reference memory task (Figures 4D,E). Figures 4F,G showed that treatment with TAT-PEP significantly improved the percentage of time and distance traveled in the target quadrant for 3 months and 18 months groups. Similarly, the data for 18 months groups in the Morris water maze evaluation was presented as a 60-day treatment of TAT-PEP, because no significant difference was detected for the 30-day treatment (data not shown).

### BDNF Expression but Not NGF Is Upregulated in TAT-PEP Treated Mice

We explored the potential mechanism for TAT-PEP-mediated motor and cognitive behavior enhancement by analyzing the expression of brain-derived neurotrophic factor (BDNF) and NGF in the brain of 18 months mice. Both BDNF and NGF are known to play major roles in promoting neuronal survival, neurite growth and cognitive ability (Lu et al., 2014; Lin et al., 2015). At the end of the behavioral test, the cerebral cortex, cerebellum and hippocampus were harvested and subjected to an ELISA assay. Results in Figure 5 showed that the 60-day TAT-PEP treatment significantly enhanced the level of BDNF in all three sub-regions. By contrast, no significant difference in NGF expression was seen between mice with TAT-PEP and TAT-mPEP treatment. These results suggest that the motor and cognitive behavior enhancement effect of TAT-PEP is likely mediated by BDNF.

## Discussion

As a functional receptor for the myelin inhibitors of axonal regeneration, PirB has been previously characterized in the neurological system with injuries, including spinal cord injuries (Zhou et al., 2010), optic nerve injuries (Cai et al., 2012), hypoxic-ischemic damage (Wang et al., 2012), stroke and lipopolysaccharide-induced chronic neuroinflammation (Deng et al., 2012). Across all these disease conditions, the expression level of PirB was found to be significantly elevated. In this study, we examined both the mRNA and protein levels of PirB in young (1 month), adult (3 months) and aged (18 months) mice, and found that the expression of PirB in the cerebral cortex, cerebellum and hippocampus increased with brain development (Figure 1). Our results are consistent with a previous report by VanGuilder Starkey et al. (2012) in which they found that both the mRNA and protein levels of PirB were upregulated in subregions of hippocampus in Fischer 344–Brown Norway rats with advanced aging.

The association of PirB with motor and cognitive functions has been previously reported. In a mouse model of stroke, it was found that knockout of the PirB gene enhanced corticospinal projections and motor recovery (Adelson et al., 2012). However, similar findings were not seen in mouse models of cortical injury (Omoto et al., 2010) nor spinal cord injury (Nakamura et al., 2011). Genetic deletion of PirB leads to activation of alternative axon growth inhibitory receptors, such as NgR, integrin and Ephrin 4A. It is likely that the activated alternative pathways are sufficient to compensate for the loss of PirB in cortical injury or spinal cord injury but not in stroke. In wild type animals, inhibition of PirB prior to the full activation of alternative pathways is likely able to induce significant therapeutic benefits, as seen in an induced stroke model (Li et al., 2017) and in the aging mice described in this study.

The extracellular segment of PirB consists of six extracellular Ig-like domains, D1 to D6 from the N- to the C-terminus (Takai, 2005). Among them, D1-D2 and D3-D6 have high affinities with MHCI and Nogo-66, respectively (Matsushita et al., 2011). It was previously reported that, during the process of age-related hippocampal changes, which were accompanied with cognitive decline, the expression levels of MHCI and PirB were elevated, suggesting that D1-D2 of PirB might promote cognition deficits through interaction with MHCI (VanGuilder Starkey et al., 2012). By contrast, Nogo-66 is correlated with motor behavior, thus we speculate that D3-D6 domain mainly mediates PirB-related motor deficits (Fouad et al., 2001). In this study, TAT-PEP was designed to include all of the six domains. Therefore, the effects of TAT-PEP treatment observed in this study is likely due to the interaction of TAT-PEP with both MHCI and Nogo-66.

We found that the therapeutic benefit of TAT-PEP treatment might be mediated by BDNF. BDNF is known to play an important role in the regulation of learning and memory (Komulainen et al., 2008; Cowansage et al., 2010; Lu et al., 2014; Tong et al., 2015), and can be used as a therapeutic agent to alleviate cognitive impairment (Wu et al., 2015). Recently, Raiker et al. (2010) reported that myelin inhibitors antagonize BDNF-induced signaling cascades through attenuation of Erk1/2 activation. This result suggests that myelin inhibitors and their receptors, such as PirB, may coordinate structural and functional neuronal plasticity in CNS health and disease through regulation of BDNF signaling, and thus, could be targeted for improvement of neurological functions. This hypothesis is well supported by the findings described in this study.

In summary, we found that PirB is correlated with age and might be a promising molecular target for modulation of motor and cognitive dysfunctions. Our results suggest TAT-PEP as a promising therapeutic agent in the future. Of course, TAT-PEP only antagonizes the extracellular segments of PirB and partly inhibits the signaling pathway of axon regeneration. Besides PirB, there are still other receptors such as NgR, integrin and Ephrin 4A that exert similar functions. Therefore, combined antagonists targeting all the receptors will be a more expected treatment strategy.

## Author Contributions

X-CGou and J-BZ designed the experiments. Y-JM, NG, Z-HZ, X-CGao, R-SZ and M-YS performed the experiments. HC and X-LW did the data analyses. Y-JM, X-CGou and J-BZ wrote the article, with the help of the co-authors.

## Conflict of Interest Statement

The authors declare that the research was conducted in the absence of any commercial or financial relationships that could be construed as a potential conflict of interest.
